# A cell-based screening method using an intracellular antibody for discovering small molecules targeting the translocation protein LMO2

**DOI:** 10.1126/sciadv.abg1950

**Published:** 2021-04-09

**Authors:** Nicolas Bery, Carole J.R. Bataille, Angela Russell, Angela Hayes, Florence Raynaud, Sabine Milhas, Sneha Anand, Hanna Tulmin, Ami Miller, Terence H. Rabbitts

**Affiliations:** 1 Weatherall Institute of Molecular Medicine MRC Molecular Haematology Unit, University of Oxford John Radcliffe Hospital, Oxford OX3 9DS, UK.; 2 University of Oxford Chemistry Research Laboratory, University of Oxford, Oxford OX1 3TA, UK.; 3 Institute of Cancer Research, 15 Cotswold Road, Sutton, London SM2 5NG, UK.

## Abstract

Intracellular antibodies are tools that can be used directly for target validation by interfering with properties like protein-protein interactions. An alternative use of intracellular antibodies in drug discovery is developing small-molecule surrogates using antibody-derived (Abd) technology. We previously used this strategy with an in vitro competitive surface plasmon resonance method that relied on high-affinity antibody fragments to obtain RAS-binding compounds. We now describe a novel implementation of the Abd method with a cell-based intracellular antibody–guided screening method that we have applied to the chromosomal translocation protein LMO2. We have identified a chemical series of anti-LMO2 Abd compounds that bind at the same LMO2 location as the inhibitory anti-LMO2 intracellular antibody combining site. Intracellular antibodies could therefore be used in cell-based screens to identify chemical surrogates of their binding sites and potentially be applied to any challenging proteins, such as transcription factors that have been considered undruggable.

## Teaser

A cell-based screening uses intracellular antibodies to select compounds targeting the chromosomal translocation protein LMO2.

## INTRODUCTION

Intracellular antibodies are a class of reagent that bind targets in the cellular environment (*1*, *2*). They are tools that can be functionalized with effector warheads such as cell death (*3*, *4*), proteasome components for targeted-protein degradation (*5*, *6*), or be directly used as inhibitors of protein activities (*7*, *8*). Furthermore, protein-protein interaction (PPI) can be attenuated by intracellular antibodies, which is an important tool in target validation (*9*, *10*). PPIs are a target class that has been challenging to convert to therapeutics with small-molecule inhibitors because they not only are usually composed of relatively large interaction surfaces involving several binding hotspots but also usually lack a well-defined binding site (or pocket) (*11*). Nevertheless, PPIs can be efficiently inhibited by macromolecules such as intracellular antibody fragments [e.g., single-chain Fragment variable (scFv) (*12*–*14*) or intracellular domain antibodies (iDAbs) (*9*, *10*, *15*)] and other intracellular antibody–like formats (*16*, *17*). The advantages of intracellular antibody–based reagents are that the natural properties of antibodies such as their high affinity and specificity can be exploited. Furthermore, their relatively quick selection processes with methods such as intracellular antibody capture (*13*) allow their use to investigate their effects on a target disease in relevant preclinical models (target validation) (*9*, *10*, *15*). While the aim of using intracellular antibodies as drugs in their own right [termed macrodrugs (*18*)] is still being developed, the small size of the iDAb interaction surface with target antigens has been explored as a template for small-molecule surrogates in a method called Abd technology (antibody-derived compound technology) (*19*). The initial Abd selection was carried out as a biochemical assay. We used a competitive surface plasmon resonance (cSPR) method to select compounds from a fragment library overlapping the antibody-binding site on HRAS^G12V^ (*19*). This selection method yielded RAS-binding fragment hits that were developed by structure-guided design to nanomolar interacting compounds that inhibited RAS-effector interactions (*19*). However, the cSPR Abd depends on favorable binding properties of the intracellular antibody with its target (very high affinity, high *K*_on_, and low *K*_off_), on the selected compounds having advantageous properties in cellular uptake and on the feasibility to express and purify the recombinant protein of interest. Consequently, new versatile methods that enable the rapid discovery of compounds targeting challenging proteins, such as the product of chromosomal translocations or transcription factors, would be valuable as these have been considered to be extremely difficult drug targets.

In this study, we developed a novel approach to development of Abd compounds with a cell-based screening method. With this new Abd technique, the interaction of target protein with iDAb was monitored by bioluminescence resonance energy transfer 2 (BRET2) signal. We applied this method to a challenging protein to target, the LIM domain only protein 2 (LMO2) that is activated by chromosomal translocations t(11;14)(p13;q11) and t(7;11)(q35;p13) in T cell acute lymphoblastic leukemia (T-ALL) (*20*). In addition, LMO2 is overexpressed in more than 50% T-ALL (*21*) and is not expressed in normal T cells (*22*). We have previously used an intracellular VH, VH576, binding to LMO2 (hereafter named iDAb LMO2) to validate the LMO2 protein target in T-ALL by showing that T cell tumors do not grow when LMO2 is blocked (*9*). The mechanism of this inhibition is that the iDAb binds to LMO2, causing a stable structure that precludes the PPI with its natural partners (*23*). We have used this anti–LMO2 iDAb as a tool for the development of a cell-based chemical library screening method to select compounds that bind to the same interface of LMO2 as the iDAb (i.e., the iDAb combining site). We established a BRET2-based LMO2 biosensor involving the interaction of LMO2 with a mutant of the LMO2 iDAb where the affinity had been reduced (dematured) by mutation of the VH complementary determining regions (CDRs). The purpose is to lower the interaction sufficiently to facilitate compounds from a chemical library to inhibit LMO2-iDAb interaction. The screening identified a chemical hit series that binds to LMO2 and interferes with iDAb binding in cells. The chemical matter was subjected to a structure-activity relationship (SAR) study to monitor the new analogs’ potency to interfere with LMO2 PPI in cells. Our study shows a further implementation of intracellular antibodies as starting points toward the selection of small molecules and, in this case, the basis of future inhibitors of the chromosomal translocation-activated protein, LMO2. Therefore, using antibodies in a drug discovery program is a new practical use with a huge potential, particularly for difficult-to-target proteins.

## RESULTS

### Establishing a BRET-based LMO2-iDAb biosensor for a small-molecule screen

We previously described the use of a high-affinity intracellular antibody binding to RAS protein in a cSPR screening of a chemical library screen (*19*). The method relies on high-affinity interaction between antibody and antigen on the SPR chip to select Abd compounds. Because the interaction affinity of the anti–LMO2 iDAb for LMO2 is the nanomolar range, rather than picomolar as the anti-RAS, and LMO2 can be expressed in *Escherichia coli* only when in complex with the LID domain of LIM domain binding 1 (LDB1) (*24*) or with the iDAb (*23*), the implementation of the cSPR Abd method to LMO2 could be challenging.

Therefore, an alternative approach was designed using a cell-based screening method for iDAb surrogates. Such a cell-based screen for compounds that inhibit PPIs requires an assay that generates a signal from the PPI but which does not occur via a high-affinity interaction because initial chemical hits would be expected to be weak binders. Accordingly, we engineered a BRET-based LMO2-iDAb LMO2 biosensor based on the strategy of RAS biosensors (*25*). We used structural data from LMO2-iDAb LMO2 complex (*23*) to optimize the proximity of donor and acceptor moieties. The donor moiety RLuc8 was fused at the C-terminal end of LMO2 and the green fluorescent protein 2 (GFP^2^) acceptor molecule to the N-terminal end of the iDAb LMO2. The interaction between LMO2-RLuc8 and GFP^2^–iDAb LMO2, the lower affinity GFP^2^–iDAb LMO2_dm_ [a dematured iDAb LMO2 (*25*)], or the nonrelevant GFP^2^–iDAb RAS (*10*) (hereafter named iDAb control or iDAb Ctl) was tested by BRET donor saturation assays (fig. S1A). These data demonstrate that the dematuration mutagenesis has lowered the affinity of the iDAb LMO2_dm_, since there is 10-fold increase in BRET_50_ (an approximation to the relative affinity of the acceptor for the donor protein) of iDAb LMO2_dm_ compared to iDAb LMO2 (0.44 versus 0.03, respectively; see fig. S1A). The specificity of these interactions was assessed with a BRET competition assay in which an untagged competitor (iDAb LMO2) or a nonrelevant competitor (iDAb Ctl) were expressed with either the BRET pairs LMO2-iDAb LMO2 (fig. S1B) or LMO2-iDAb LMO2_dm_ (fig. S1C). The competitor iDAb LMO2 decreased LMO2-iDAb LMO2 interaction in a dose-dependent manner but only to ~65% at the highest dose of competitor (fig. S1B). Accordingly, iDAb LMO2 competed the lower-affinity LMO2-iDAb LMO2_dm_ interaction with a stronger inhibition at its highest dose (~80%; fig. S1C), and the expression of these proteins was not altered (fig. S1D). These data suggest that the affinity of the iDAb LMO2_dm_ needed to be further decreased to be used in a screening assay where the binding strength of initial hits was likely to be low.

**Fig. 1:**
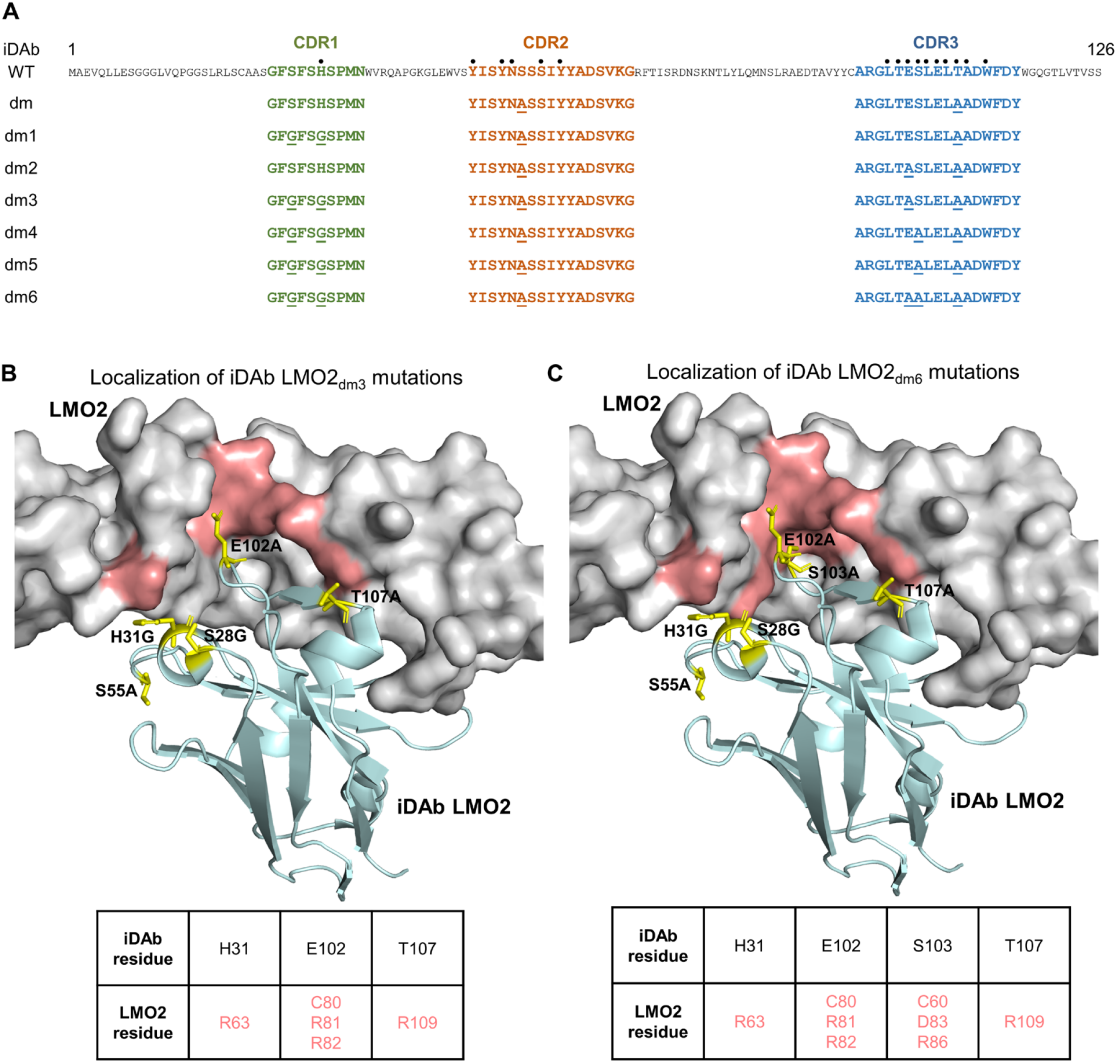
Localization of iDAb LMO2 mutations and the corresponding affected amino acids on LMO2 structure. The crystal structure of LMO2-iDAb PDB 4KFZ was used to simulate the mutation effect for the dematuration of the anti–LMO2 iDAb. (**A**) Amino acid sequence alignment of the original iDAb LMO2 [wild type (WT)] and the various iDAb mutants (iDAb LMO2_dm(1–6)_). The framework is in black, and the CDRs regions are colored in green (CDR1), orange (CDR2), and blue (CDR3). Amino acids mutated in iDAb LMO2_dm(1–6)_ are underlined, and the black dots indicate the amino acids within the CDRs making contact with LMO2 (*23*). (**B**) Localization of iDAb LMO2_dm3_ mutations (S28G, H31G, S55A, E102A, and T107A) are shown in yellow on the parental iDAb LMO2 structure, with the affected LMO2 residues shown in red on LMO2 structure (displayed in gray). (**C**) Localization of iDAb LMO2_dm6_ mutations (S28G, H31G, S55A, E102A, S103A, and T107A) are shown in yellow on the parental iDAb LMO2 structure, with the affected LMO2 residues shown in red on LMO2 structure (displayed in gray). LMO2-iDAb LMO2 structure used is PDB 4KFZ. Each panel has a table listing the interacting amino acids of LMO2 and iDAb.

**Fig. 2:**
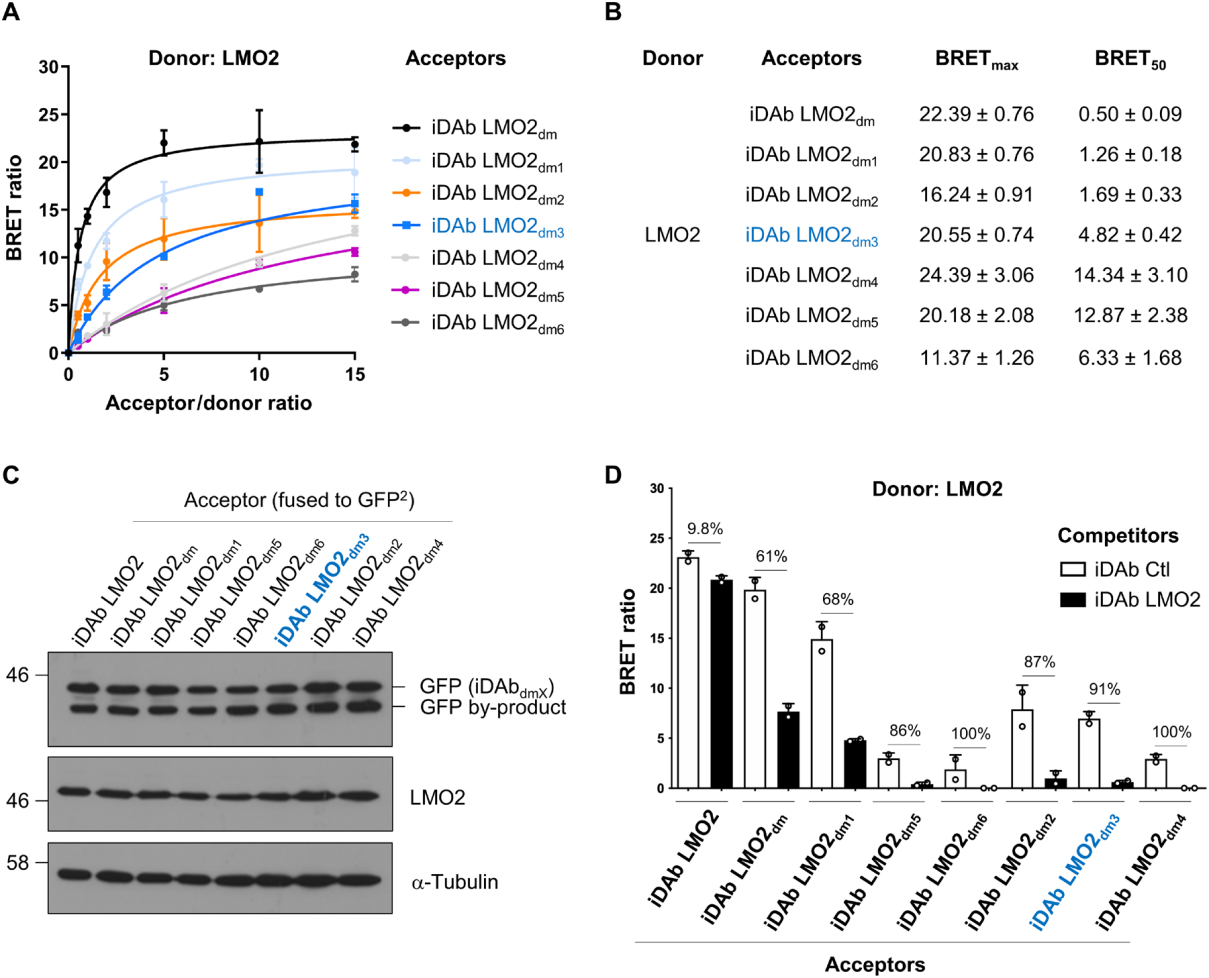
Establishing a BRET-based LMO2-iDAb biosensor amenable for a high-throughput screening of small-molecule libraries. A BRET biosensor was established for the interaction of LMO2 with the anti-LMO2 VH by titrating BRET signal for mutant VH binding to LMO2. The BRET2 assay comprises live in-cell generation of signal following interaction of a donor protein (in this case, LMO2-RLuc8) and an acceptor protein (in this case, GFP^2^–anti–LMO2 iDAb) and BRET signal (energy transfer from activated RLuc8 to GFP^2^). (**A**) BRET donor saturation assay with donor LMO2 and different mutant iDAb LMO2 acceptors, iDAb LMO2_dm_, LMO2_dm1-dm6_. (**B**) BRET_max_ and BRET_50_ values from the donor saturation curves displayed in (A). (**C**) Western blot data for the expression of the GFP^2^–iDAb LMO2 and mutants (using anti-GFP antibody) and expression of LMO2-RLuc8 (with anti-LMO2 antibody). α-Tubulin is the loading control. (**D**) BRET competition assay of LMO2-RLuc8 and the different GFP^2^–iDAb LMO2_dmx_ by expression of a nonrelevant control iDAb [anti-RAS (*10*); Ctl, white bars] or unmutated iDAb LMO2 (black bars) as competitors. This competition is performed at the lowest dose of competitor (i.e., 0.1 μg, see Materials and Methods). The percentage inhibition by iDAb LMO2 compared to iDAb Ctl is displayed. The iDAb LMO2_dm3_ mutant choose for the cell-based screening assay is colored in blue. Each experiment was performed twice. Where error bars are presented (A and D), they correspond to mean values ± SD of biological repeats.

We have used a dematuration method to decrease iDAb affinity based on CDR sequences (*26*) such as it enabled an alpha-screen of RAS^G12V^-binding compounds and analysis of in vitro–derived RAS-binding Abd compounds (*27*). On the basis of the LMO2-iDAb LMO2 structural information (*23*), we introduced additional mutations on the CDRs of iDAb LMO2_dm_ that would affect the interaction between key amino acids from the iDAb and LMO2 with alanine or glycine substitution while still retaining specific binding (Fig. 1A). Hence, we constructed six mutants named iDAb LMO2_dm1_ to iDAb LMO2_dm6_ [DNA and protein sequences shown in fig. S2 (A to H)]. Most of the modifications on the iDAb LMO2 affected its binding around the hinge region of LMO2 (Fig. 1, B and C). Next, we tested them in BRET donor saturation assays (Fig. 2A). Each of the iDAb LMO2 mutants (dm1 to dm6) had a decreased BRET_max_ value (an approximation for the total number of complex LMO2/iDAb and the distance between the donor and the acceptor within the dimer) and an increased BRET_50_ value compared to the template iDAb LMO2_dm_ (Fig. 2B). This suggested an overall decreased affinity of the dematured iDAbs toward LMO2, and the mutations did not affect their expression (Fig. 2C). Last, we performed a BRET competition experiment with each mutant (Fig. 2D) to determine the optimal dematured iDAb for the chemical library screen. The competition data with iDAb LMO2_dm3_ showed that it was the best mutant as its interaction with LMO2 was almost completely inhibited by iDAb LMO2 (~90%) while it retained a relatively high BRET signal (Fig. 2D). Therefore, we chose this mutant for a cell-based high-throughput screening of small molecules.

### HTS for inhibitors of LMO2-iDAb_dm3_ interaction

**Fig. 3:**
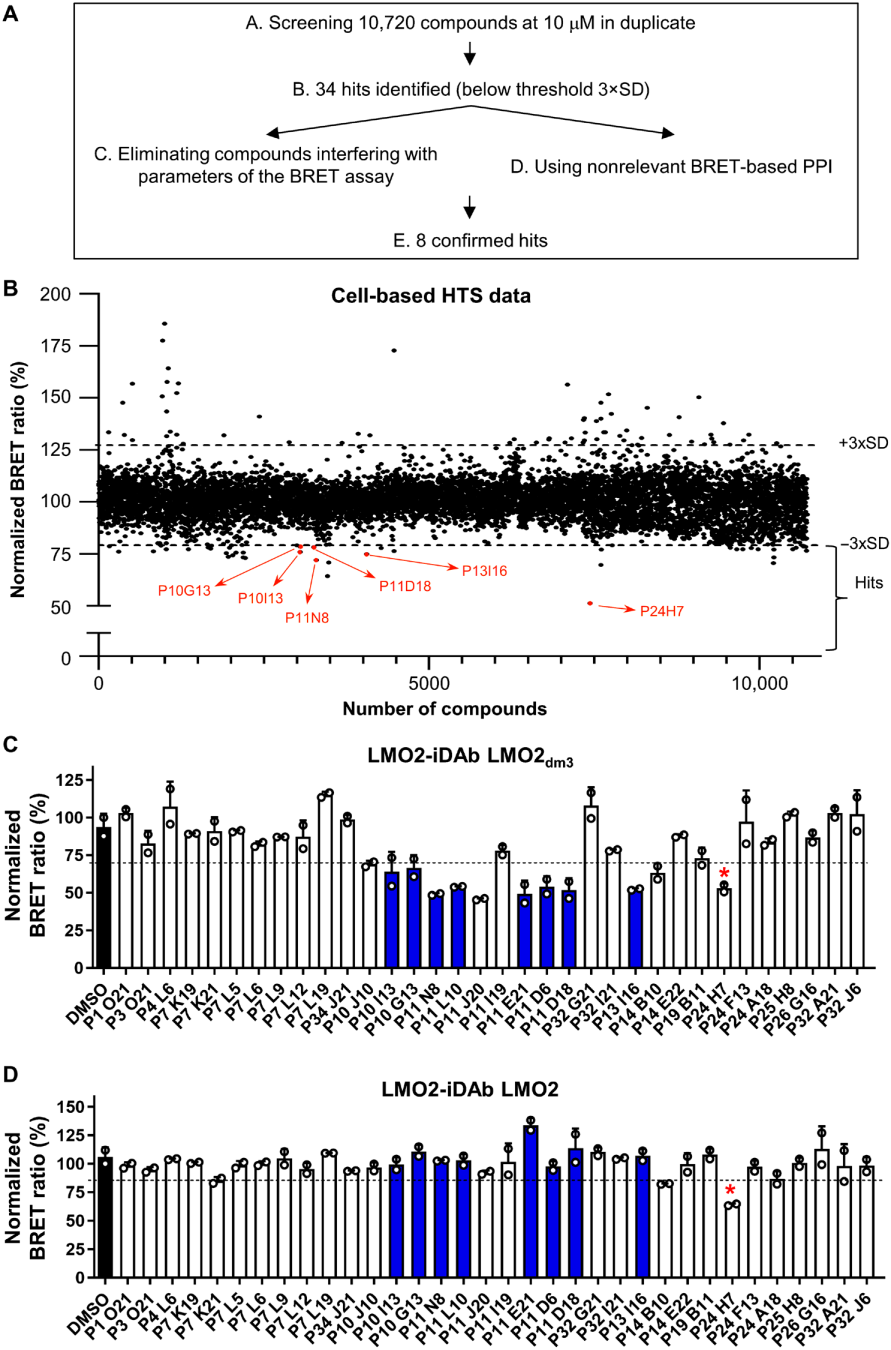
Cell-based high-throughput screening for inhibitors of LMO2-iDAb LMO2 PPI. A high through-put screen of a diverse compound library was conducted using the LMO2-VH BRET assay. (**A**) The scheme for the cell-based HTS is shown where a diverse chemical library of 10,720 compounds was screened using a BRET cell assay to determine diminution of signal generated by interaction of LMO2-RLuc8 and GFP^2^-iDAb_dm3_. (**B**) Scatter plot of the normalized BRET signal from 10,720 compounds tested at 10 μM. Thirty-four compounds (primary hits) caused inhibition of BRET signal below a cutoff of three times the SD (minus, −3×SD) of the DMSO BRET signal (*28*). Some primary hits are pinpointed in orange. (**C** and **D**) Confirmation of inhibition of BRET signal from LMO2 interaction with iDAb LMO2_dm3_ (C) and for interaction of LMO2 with unmutated iDAb (D). Eight hits (depicted by blue bars) were confirmed to decrease LMO2-iDAb LMO2_dm3_ signal by at least 3×SD of the BRET signal with DMSO control (i.e., DMSO BRET signal ± 3×SD: 12.2 ± 3.6, threshold set at 8.6 and shown with the dotted line) without affecting LMO2-iDAb LMO2 interaction (i.e., DMSO BRET signal ± 3×SD: 30.3 ± 3.4, threshold set at 26.9 and shown with the dotted line). P24H7 compound highlighted with a red asterisk is an example of compound that was not pursued further as it affects both iDAb LMO2_dm3_ and iDAb LMO2 interaction with LMO2. Experiments in (C) and (D) were performed twice. Error bars presented in (C) and (D) correspond to mean values ± SD of biological repeats.

We exploited the robustness and scalability of our cell-based BRET LMO2-iDAb LMO2_dm3_ interaction assay in a high-throughput screen (HTS) to identify compounds that inhibit this interaction. We screened a library of 10,720 small molecules assembled from BioFocus and ChemBridge sources (see Materials and Methods). The flowchart of the HTS is described in Fig. 3A. Human embryonic kidney (HEK) 293T cells were transfected on day 1 with plasmids expressing LMO2-RLuc8 and GFP^2^–iDAb LMO2_dm3_, and 24 hours later, compounds were added to 10 μM and the BRET signals were determined after a further 24 hours. The entire screen was done in duplicate plates. Ninety-nine compounds modulated LMO2-iDAb LMO2_dm3_ BRET interaction within both duplicates using a cutoff of 3×SD from dimethyl sulfoxide (DMSO) controls (*28*) in order that the number of hits could readily be handled in secondary assays (Fig. 3B). Sixty-five compounds potentiated, and 34 primary hits inhibited LMO2-iDAb LMO2_dm3_ interaction. The 65 compounds might favor the interaction between LMO2 and iDAb LMO2_dm3_ or modify the complex in a way that brings the GFP^2^ and Rluc8 moieties closer or in an orientation that is more permissive for energy transfer. It is also possible that the increase of signal is due to an increase of GFP^2^ signal (e.g., if a compound emits in the GFP^2^ channel). We focused on the 34 compounds as we sought to identify inhibitors of LMO2-iDAb LMO2_dm3_ interaction. These were retested using the original BRET assay to confirm inhibition of signal. Because selected compounds should be weak binders, we also used BRET-based assay between the strong interaction LMO2 and unmutated iDAb LMO2 to eliminate nonspecific compounds that could bind to key residues of the iDAb, such as residues L104/E105/L106 that were previously shown essential for LMO2 binding by mutagenesis (Fig. 3, C and D) (*23*). In addition, initial hits affecting RLuc8 luminescence or intrinsic GFP^2^ fluorescence by greater than twofold were not considered, further including many potent primary hits such as P24H7 (fig. S3, A and B). This rescreen confirmed eight inhibitors of LMO2-iDAb LMO2_dm3_ interaction that corresponded to ≈25% of the primary hits (fig. S3, C and D). The eight compounds were lastly tested with a nonrelevant BRET-based interaction assay (MAX bHLH-CMYC bHLH) to provide further confirmation of specific interaction with LMO2 (fig. S3E). The chemical structures of the selected hits show that the compounds belong to a family that can be divided into two subfamilies because of their chemical similarities. The main difference is the presence of either a five- or seven-membered ring in each subfamily (fig. S3, C and D, respectively).

### SAR study of Abd compounds

**Fig. 4:**
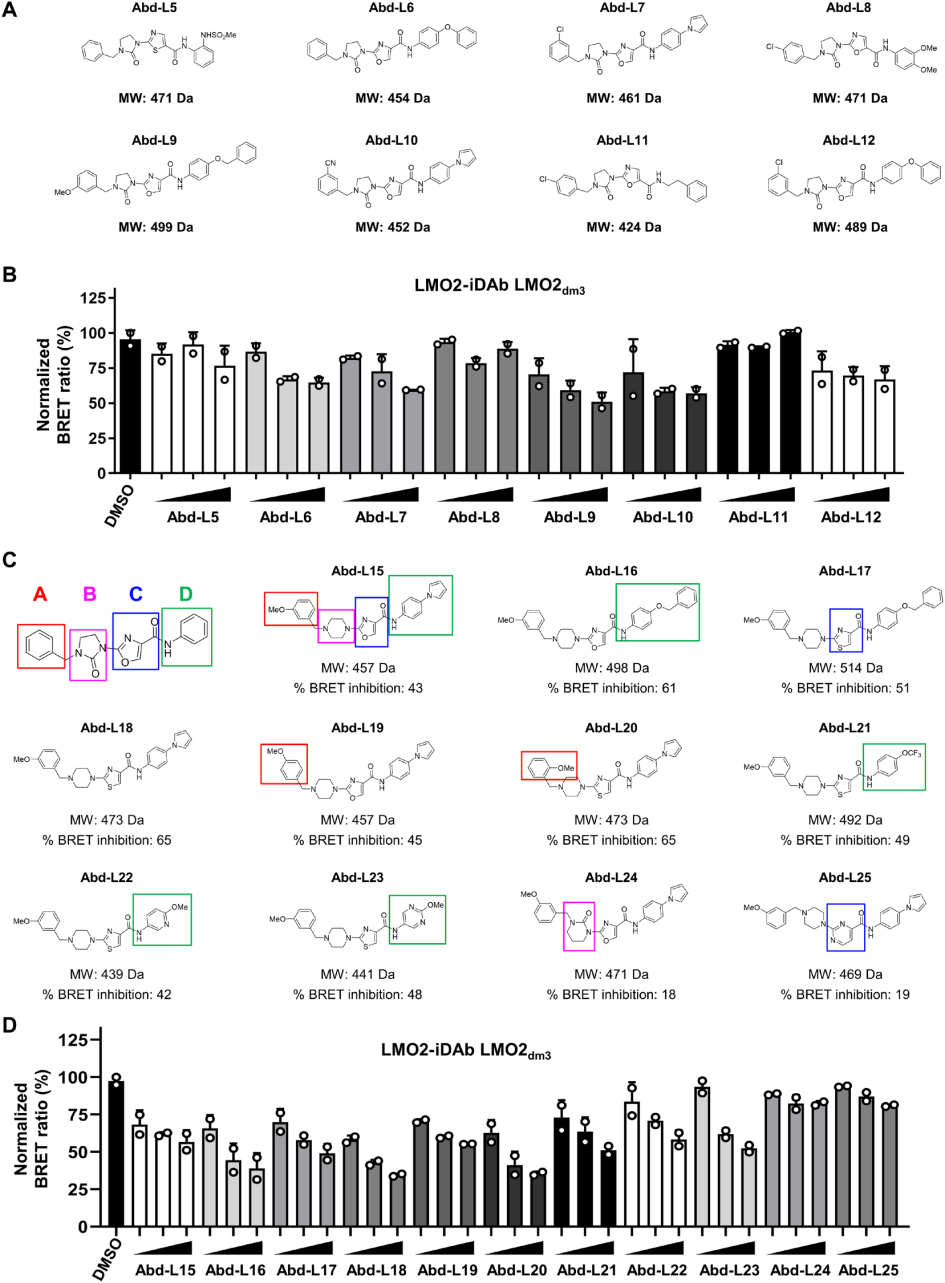
Structure-activity relationship of Abd LMO2-binding compounds. Hits identified from the HTS library screen were classified and a family of related compounds identified as the basis of SAR analysis. The chemical structures of the hit matter from the BRET screen were examined and a family of compounds was identified. (**A**) Chemical structures of the 8 re-synthesized hits (Abd-L5 to Abd-L12) with their respective molecular weight (MW). (**B**) Dose response inhibition of LMO2-iDAb LMO2_dm3_ interaction by compounds Abd-L5 to Abd-L12 (concentration range: 1, 10, and 20 μM). (**C**) SAR study of Abd-L9 compound as template. The compound was divided into four substituents (named A to D) that were substituted by various other chemical groups to give new compounds. The colored boxes (red, pink, blue, and green) represent the different moieties substituted in the SAR study in positions A, B, C, and D, respectively. Structures are shown of representative compounds from Abd-L15 to Abd-L25. The new compounds were tested by BRET assays with LMO2-iDAb LMO2_dm3_ interaction. The different SAR-derived compounds are shown with their MW and the percentage of BRET inhibition of the interaction LMO2-iDAb LMO2_dm3_ at 20 μM including Abd-L24 and Abd-L25 modified on their positions B and C, respectively, which do not affect the BRET interaction LMO2-iDAb LMO2_dm3_. (**D**) Dose response effect of the Abd-L15 to Abd-L25 compounds on LMO2-iDAb LMO2_dm3_ interaction (Abd concentration used: 5, 10, and 20 μM). Experiments in (B) and (D) were performed twice. Error bars presented in (B) and (D) correspond to mean values ± SD of biological repeats.

Samples of the five-membered ring-containing hits (fig. S3C) were prepared following literature methods. However, we found that the core underwent a rearrangement to the five-membered when attempting to synthesize samples of seven-membered ring-containing compounds (fig. S3D). Therefore, we synthesized the corresponding five-membered ring analogs, which were named Abd-L5 to Abd-L12 (Fig. 4A). We tested these analogs in a dose-response BRET assay to verify their ability to bind LMO2 and their potency in cells (Fig. 4B and fig. S4A). Three of the analogs, Abd-L5, Abd-L8, and Abd-L11, were unable to inhibit LMO2-iDAb LMO2_dm3_ interaction (Fig. 4B). Hence, the most potent inhibitor, Abd-L9, was used as a template for a SAR study (Fig. 4C).

The different moieties of Abd compounds were divided into four substituent groups—namely, benzyl (position A), imidazolidinone (position B), oxazole (position C), and aniline (position D)—and were modified systematically (Fig. 4C). Representative analogs are shown (Abd-L13 to Abd-L25; Fig. 4C and fig. S4, B to E). In position A, most substituted benzyl groups were found to be well tolerated (see red boxes in Fig. 4C). Notably, the methoxy group could be placed in ortho, meta, or para positions on the benzyl ring (see red boxes on Abd-L15, Abd-L19, and Abd-L20) with minimal effect on the BRET inhibition potency of derivatives (Fig. 4D and fig. S4, C to E). At position D, it was found that a large array of substituted anilines and benzyl amines were also well tolerated (green boxes in Fig. 4C and BRET data in Fig. 4D and fig. S4, C to E).

Modifications to positions B and C had more substantial effect on the potency of the analogs (Fig. 4, C and D). In position B, any replacement of the imidazolidinone was found to bring a loss of activity (see pink box on Abd-L24; Fig. 4C) apart from the corresponding piperazine (see pink box on Abd-L15 in Fig. 4C). Because of the potential chemical instability of the imidazolidinone, the lower yields, and a large number of side products during synthesis, the piperazine moiety was used in further SAR investigations, eradicating those issues. In position C, it was found that only the 2,4-substituted-thiazole (Fig. 4C, blue box on Abd-L17) and 2,4-substituted-oxazole (Fig. 4C, blue box on Abd-L15) were tolerated as a core and the corresponding 2,5-substituted heterocycles and different heterocycle such as pyrimidine (see blue box on Abd-L25 in Fig. 4C as example) led to a loss of activity (Fig. 4D). These data suggested that the B and C positions are important for the interaction of the compounds with LMO2, while position A and D could be modified to add new functional groups.

We also tested Abd-L9 and some analogs in a parallel artificial membrane permeability assay (PAMPA) and Abd-L9 in a Caco-2 permeability assays (fig. S5, A and B). This showed that the compounds were permeable through a synthetic membrane (PAMPA) or into cells (Caco-2) as would be expected from compounds derived from cell-based screens. Abd-L9 showed the best properties in the PAMPA compared to the analogs and a low transport but low efflux ratio in the Caco-2 assay (fig. S5, A and B). These results suggested that while Abd-L9 enters cells with a relatively low efficiency, it is not actively exported from the cells (low efflux ratio).

### Abd compounds bind LMO2 in vitro

**Fig. 5:**
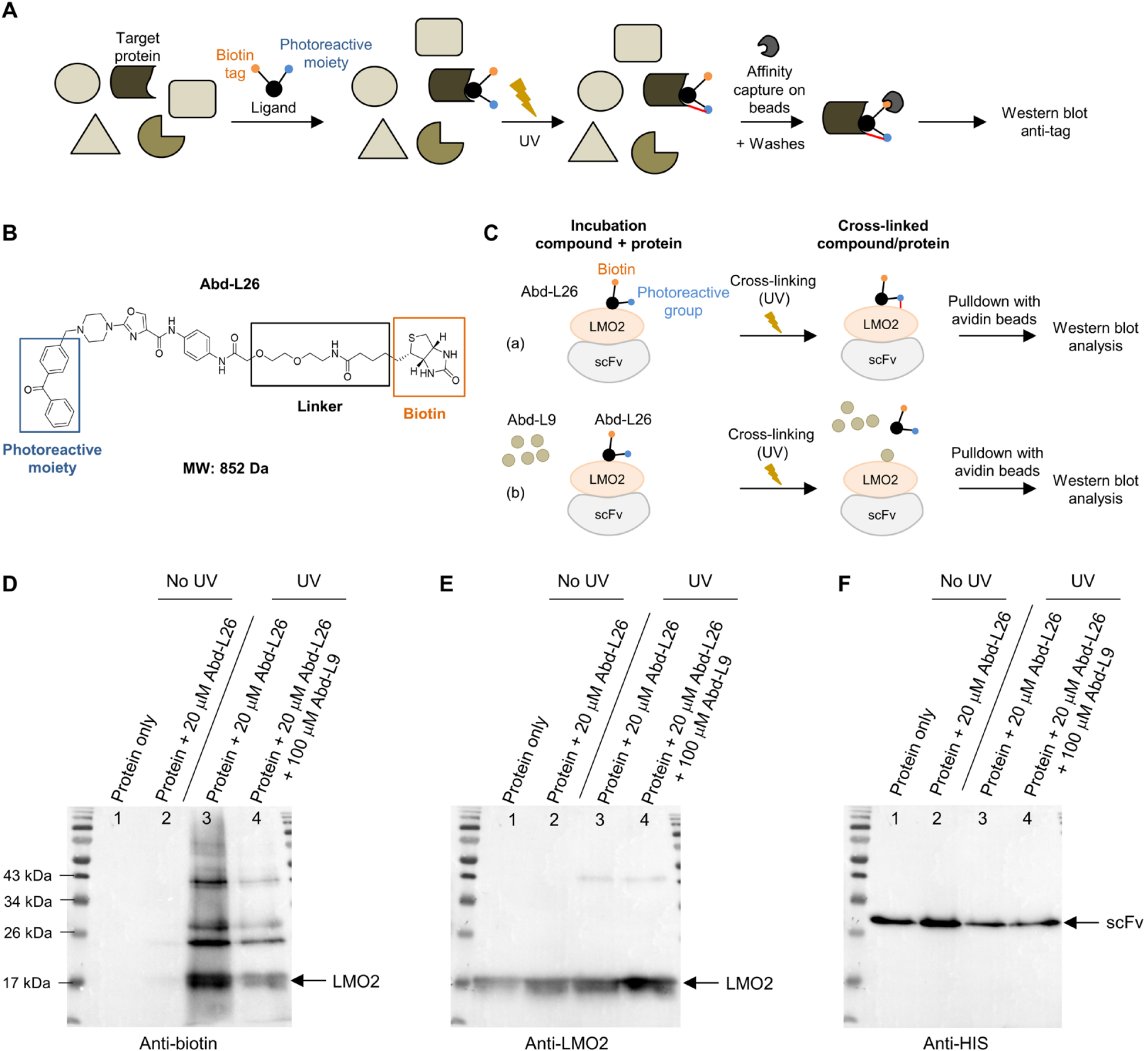
Abd compounds bind to LMO2 in vitro. (**A**) General scheme of the PAL method. A cell lysate or a partially purified mixture of proteins containing the target protein are incubated with a functionalized ligand. This ligand (black circle) has a tag (orange circle) and a photoreactive moiety (blue circle). The mixture is irradiated with UV to induce the cross-linking between the photoreactive group and the protein bound by the ligand (red line). Cross-linked complexes are purified by affinity capture on beads (via the tag) and washed to remove nonspecific proteins. Washed beads are denatured and loaded on a SDS–polyacrylamide gel electrophoresis, and the membrane is probed with an anti-tag antibody. (**B**) Chemical structure of Abd-L26 built on Abd-L15/16 template, with a benzophenone photoreactive moiety, a linker, and a biotin tag and designed for PAL to LMO2 protein. (**C**) Illustration of the LMO2/Abd-L26 PAL experiment. In (a), Abd-L26 compound is incubated with scFv-LMO2 partially purified complex. After UV cross-linking, an affinity pulldown is performed with avidin beads that capture the biotin moiety of Abd-L26 and the proteins pulled down are analyzed by Western blot. (b) A competition experiment was performed the same way but with the addition of an excess of competitor, Abd-L9, which should reduce the cross-linking of LMO2 by Abd-L26 if the interaction is specific. (**D** to **F**) Pulldown of scFv-LMO2 recombinant protein by Abd-L26 with avidin beads. The protein was either incubated alone (lane 1) or with 20 μM Abd-L26 (lane 2) without UV treatment. The protein was also incubated with 20 μM Abd-L26 alone (lane 3) or with 100 μM Abd-L9 as competitor (lane 4) treated by UV light. The beads were washed, and a Western blot showed the quantity of cross-linked LMO2 on the beads with an anti-biotin (D), anti-LMO2 (E), and an anti-HIS antibody (F).

The LMO2 Abd compounds were subsequently verified using an in vitro orthogonal assay. We used the photoaffinity labeling (PAL), a powerful technique to study protein-ligand interactions in cell lysate or with (partially) purified proteins (*29*) (Fig. 5A). PAL corresponds to the use of a chemical probe added on a ligand that can then covalently bind to its target in response to activation by ultraviolet (UV) light (Fig. 5A) (*30*). In addition, a tag moiety is added on the ligand that allows the capture of the covalent ligand-protein complex by affinity beads before detection by Western blot (Fig. 5A). The extensive SAR data on the LMO2 Abd compounds suggested attachment sites on the parent ligand. We added a benzophenone photoreactive group in place of the benzyl substituent (position A) and a linker with a biotin tag in position D (designated Abd-L26; Fig. 5B). To test whether the addition of the photoreactive group on the Abd compound would modify its potency, and because the biotin moiety may render compounds cell impermeable (*29*), we tested the ability of a precursor compound, Abd-L27 (fig. S6A), to inhibit LMO2-iDAb LMO2_dm3_ interaction in BRET assay. We observed that Abd-L27 retained its inhibitory potency in BRET (fig. S6, B and C) and thus maintains its ability to bind to LMO2.

The analysis of PAL technique requires soluble, recombinant LMO2 in order that it has the Abd-L compound-binding site accessible. We carried out a phage display screen of scFvs with LMO2-LID protein antigen and obtained scFv that binds LMO2 and can be coexpressed in *E. coli*. By using the partially purified scFv-LMO2 dimer, the PAL technique was performed after inducing cross-linking of Abd-L26 to the scFv-LMO2 complex with UV light for photocrosslinking (illustrated in Fig. 5C). The Abd-L26 in the complex was isolated by interaction of the biotin moiety with avidin beads and the protein analyzed by Western blot with either anti-biotin antibody (Fig. 5D), anti-LMO2 antibody (Fig. 5E), or anti-HIS tag (Fig. 5F). The pulldown data show that protein is only cross-linked when the mixture is treated with UV light, and we observed a protein (Fig. 5D, lane 3) coincident with the size of LMO2 (Fig. 5E). In addition, the recovery of biotinylated LMO2 was inhibited by incubating the protein with Abd-L26 (PAL) in the presence of 5× concentration of Abd-L9 competitor (Fig. 5D, lane 4), confirming a specific binding of the compound on LMO2. The anti-biotin antibody showed that the biotinylated proteins specifically bound to the beads through the PAL Abd-L26 compound, while the anti-LMO2 and anti-HIS antibodies show nonspecific binding of proteins to the beads. We noted that the recombinant LMO2 and the scFv had a tendency to associate nonspecifically with avidin agarose beads used for the pulldown without UV cross-linking (see lanes 1 and 2, Fig. 5, E and F). This may be due to partial denaturation of the proteins during the PAL incubation and explains the apparent partial inability of Abd-L9 to compete the PAL compound (Fig. 5F, lane 4 versus lane 3).

### Activity of LMO2 Abd compounds in cells

We tested the specificity and potency of Abd-L compounds in cells by using dose-response BRET assays on different LMO2 PPI. This included LMO2 interaction with the unmutated iDAb and the iDAb_dm3_, with its natural partner proteins LDB1 and TAL1 (together with E47) (*31*) and with a nonrelevant control PPI, which is the interaction of the bHLH regions of CMYC with MAX. We first tested the direct interaction LMO2 with TAL1 by a BRET donor saturation assay (fig. S7A), but this interaction is weak and gave a high BRET_50_ value. We added individually the partner proteins involved in the LMO2 complex (*31*) and found that coexpression of E47, a heterodimerization partner of TAL1, increased the relative affinity of LMO2-TAL1 and that the addition of LDB1 gave the strongest binding between LMO2 and TAL1 (fig. S7A; see decreasing BRET_50_ values, from 12.6 to 1). We also developed the BRET pairs LMO2-LDB1 (fig. S7B) and the nonrelevant interaction of MAX bHLH with CMYC bHLH (fig. S7C). Last, the specificity of these three interactions was tested, with BRET competition assays, by coexpressing nontagged versions of iDAb Ctl or iDAb LMO2 in the BRET assay cells. iDAb LMO2 inhibited BRET signal from LMO2-TAL1 + E47 (fig. S7D) and from LMO2-LDB1 (fig. S7E) but not from MAX-CMYC interaction (fig. S7F).

**Fig. 6:**
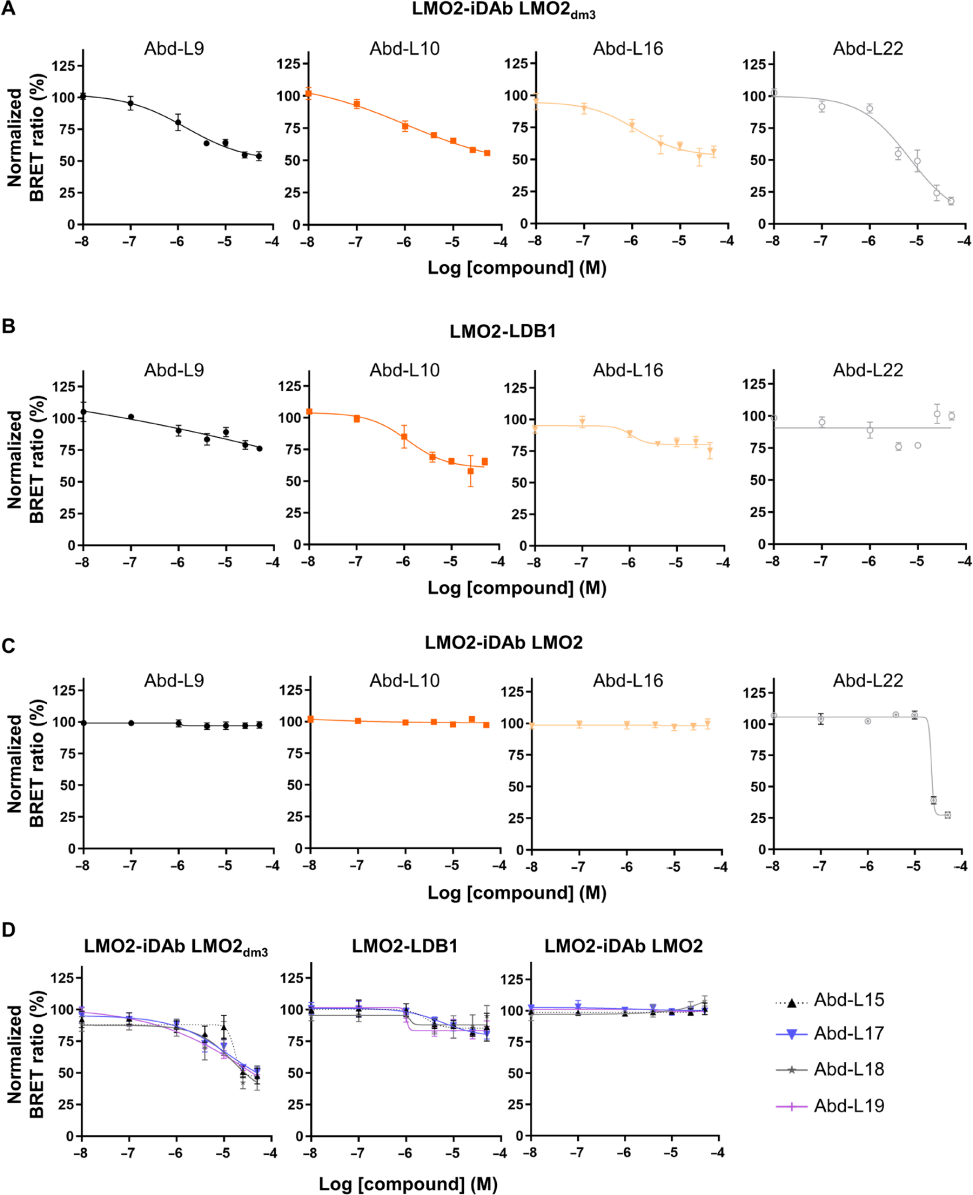
Evaluation of LMO2-binding Abd compound potency in cells. The potency and specificity of the LMO2 Abd compounds was evaluated in dose-response BRET assays. (**A** to **C**) In BRET assays, Abd-L9, Abd-L10, Abd-L16, and Abd-L22 compounds were assessed in dose inhibition responses for (A) LMO2-iDAb LMO2_dm3_, (B) LMO2-LDB1, and (C) LMO2-iDAb LMO2 (unmutated iDAb) BRET interactions. (**D**) Dose-response assays of Abd-L15, Abd-L17, Abd-L18, and Abd-L19 with LMO2-iDAb LMO2_dm3_, LMO2-LDB1, and LMO2-iDAb LMO2 BRET interactions. Each experiment was performed twice. Error bars presented in (A) to (D) correspond to mean values ± SD of biological repeats.

We assessed the anti–LMO2 Abd compounds in BRET dose-response assays with the various BRET assays (Fig. 6, A to D, and fig. S7, G and H). None of the compounds inhibited LMO2-iDAb LMO2_dm3_ BRET by more than 40 to 50%, with the exception of Abd-L22 (~85%). However, we found that Abd-L9, Abd-L10, and Abd-L16 had the best relative median inhibitory concentration (IC_50_) for the interaction LMO2-iDAb LMO2_dm3_ at around 1 μM (Fig. 6A and table S1) whether or not the compound contained imidazolidinone substituents (Abd-L9 and L10) or a piperazine substituent (Abd-L16). The other compounds tested showed relative IC_50_ values ranging from just more than 7 μM to nearly 50 μM for Abd-L19 (Fig. 6, A and D, and table S1). When this group of compounds were assayed with LMO2-LDB1 BRET, little effect was observed except for Abd-L10 that caused only a small inhibition (35% at the highest concentration of Abd-L10 with an IC_50_ of 1.2 μM; Fig. 6, B and D, and table S1). Testing this group of Abd-L compounds with the BRET assays for LMO2-iDAb LMO2 (unmutated iDAb) (Fig. 6C), LMO2-TAL1 + E47 (fig. S7G), or MAX bHLH-CMYC bHLH (fig. S7H) failed to show any inhibition, even at the highest concentration of compound as used throughout the series of BRET inhibition assays with the exception of Abd-L22 that inhibits LMO2-iDAb LMO2 interaction but only at high concentrations (above 25 μM; Fig. 6C).

## DISCUSSION

### Intracellular antibodies as templates for drug discovery

Intracellular antibody fragments interact with proteins at any antigenic site or where natural partner proteins are involved in PPI. This gives an opportunity using the intracellular antibody to derive compounds that overlap the antibody-binding site. When the intracellular antibody interferes directly with a PPI, rather than using the natural partner protein, the intracellular antibody can be obtained with very high affinity binding as we showed for selected compounds binding to the RAS proteins (*19*), demonstrating that this so-called undruggable target is, in fact, druggable. The same interaction site of an intracellular antibody fragment can be used for both target validation and drug screening [e.g., LMO2 or RAS targets (*15*, *23*)]. The use of intracellular antibody fragments rather than natural partner proteins as a general approach for drug discovery has several advantages based on the natural specificity and affinity of antibodies where domain antibodies have small binding areas of few residues compared to natural interactions that tend to be flat and with a large interaction surface (*32*), making them difficult to find compounds that are inhibitors (*33*). In addition, manipulating the affinity of intracellular antibody fragments, unlike natural partners, is potentially straightforward without structural data, since their CDRs, defined by primary sequence, are major actors of their interaction with their target antigen (*34*). This process is called intracellular antibody dematuration (*27*). Furthermore, intracellular antibodies can discriminate family members (isoforms or paralogs), which themselves may have common binding partners [e.g., KRAS (*16*)]. Last, the use of intracellular antibody is advantageous over the use of natural binders in cases where (i) the natural partner is not defined or (ii) multiple partners are involved and interact on different interfaces of the protein of interest, such as the complex involving LMO2 (*31*).

In contrast to the cSPR Abd method where a high-affinity antibody is needed (*19*), the measured in vitro affinity of the iDAbs for their target is not a limitation with our cell-based assay. High-affinity iDAbs could be dematured to reduce their binding for use in the cell-based assay such as in a RAS compound selection assay (*27*). Alternatively, an iDAb already with a lower affinity could be directly used in the cell-based assay without prior dematuration step, which makes this a flexible approach. Notably, we previously observed with iDAb RAS that affinity measured in vitro and by BRET assay were not in the same rank of order (*25*). iDAb RAS has an in vitro affinity of 6.2 nM, while the iDAb RAS_dm_ has an affinity around 1 μM (>160-fold difference). Nonetheless, the BRET_50_ value of iDAb RAS is 0.34, while it is 1.34 for the iDAb RAS_dm_ (>4-fold difference), showing that there is no direct affinity comparison possible between in vitro and BRET assay. While the quantity of proteins is known for in vitro measurements, it is not readily controlled for the cell-based assays, which therefore only gives a proxy for an affinity.

The data from Fig. 3D show that none of the hits were able to inhibit LMO2–unmutated iDAb LMO2 interaction supporting our dematuration process as key in the success of our screen. However, it is possible that the dematuration method could increase the probability of selecting false-positive binders by increasing the dissociation between the iDAb and its target. Furthermore, screening using the BRET assay could also introduce false-positive binders through small differences in protein expression levels of GFP^2^ fusion protein. This could partly explain why only 25% of the 34 primary hits was confirmed after retesting. Nevertheless, by performing the screening in duplicate plates, we aimed to reduce the probability of selecting false-positive binders.

The cell-based Abd assay is a more versatile method, as it could be implemented to any challenging protein that is difficult to express and/or to purify in recombinant form, such as LMO2. Actually, only a small quantity of nonpurified protein is necessary with the BRET assay to detect an interaction, while usually larger amounts of proteins are needed for cell-free assays. Last, the intrinsic advantage of cell-based assays, in which a signal is generated by the direct interaction of target with iDAb, is that the compounds already have the characteristic of cell entry, which we show here with our LMO2 Abd-L series of compounds. This property is highly relevant for the use of small molecules as drugs.

### LMO2 binding compounds derived from a cell-based BRET2 chemical library screen

Rather than being a pure drug development campaign per se, our study demonstrates a methodology for using antibodies in drug discovery, in this case against a chromosomal translocation protein LMO2, confirming the general applicability of our methods. We describe a cell-based intracellular single domain antibody-guided small-molecule selection method that allows the direct identification of compounds that bind at the same region of the iDAb. We have illustrated this approach using the T cell oncogenic chromosomal translocation protein LMO2 (*20*) with an inhibitory anti–LMO2 iDAb (*9*, *23*). We used the iDAb to screen a compound library (10,000 compounds) that bind to the T cell oncogenic protein LMO2 with the LMO2-iDAb BRET2 cell-based interaction assay. We obtained a number of initial hits, and one chemical series of which Abd-L5 to Abd-L12 were the progenitors. The difficulty with being unable to express and purify recombinant LMO2 protein alone in *E. coli* in a quantity that would enable structural analysis precludes this study at this stage. However, because our compounds can tolerate linkers and larger groups, as found out by SAR analysis, it was possible to use the PAL technology that requires addition of two nonbinding substituents. We tested a benzophenone moiety as PAL and observed that analogs bearing this group on the right- or the left-hand sides were still active, whereas the linker could only be located on the right-hand side. A compound (Abd-L26) was therefore prepared with the benzophenone photoreactive moiety on the piperazine and the biotin linked to the aniline in the para position (Fig. 5B). The cross-linking of Abd-L26 to the LMO2 protein confirmed binding to LMO2 protein in vitro, and this was inhibited by addition of the parental compound Abd-L9 (Fig. 5D). Notably, some contaminating bands appeared on the Western blots (Fig. 5D), as the scFv-LMO2 protein was only partially purified. In addition, scFv-LMO2 protein bound nonspecifically to the beads, and this may explain minimal decrease of LMO2 signal by competition of Abd-L9 with the LMO2 antibody (Fig. 5E, lanes 3 and 4). These data suggest that the chemical series is an intracellular antibody surrogate that binds to LMO2 where the anti–LMO2 iDAb contacts LMO2. The cell-based selection involved competition by the compounds for the interaction of LMO2 with a dematured iDAb (i.e., with a lower affinity), and these compounds do not influence the interaction of LMO2 with unmutated iDAb (except Abd-L22 that might behave aberrantly at high concentrations) as would be expected from their micromolar relative IC_50_.

By solving the structure of the LMO2/iDAb dimer, we showed that the iDAb modifies the LMO2 conformation, thereby impeding the interaction of partners such as TAL1 and E47 (*23*). We confirmed these data by BRET assay where the iDAb LMO2 blocks the binding of these proteins with LMO2 (fig. S7D). However, with the Abd-L compounds selected here, we could not observe an inhibition of the interaction LMO2/TAL1-E47 by BRET assay (fig. S7G), suggesting that the compounds do not substantially modify the conformation of LMO2 protein. Therefore, in the future, our aim is to extend these compounds to potentially achieve binding geography like that seen for the anti–LMO2 iDAb and that could achieve the same LMO2 stable conformation change that the iDAb causes, with the similar effects on the LMO2 protein complex function in T cell acute leukemia. Furthermore, our compounds could also be the starting point for the development of LMO2 PROTAC (proteolysis targeting chimera) degraders (*35*).

Intracellular antibody fragments or other macromolecules can be used to investigate their effects on a target disease in relevant preclinical models (target validation) (*9*, *10*, *15*, *36*). Furthermore, recurrent chromosomal translocations are abundant in all tumor types (*37*) that produce intracellular proteins that function in various cellular processes such as transcription, where PPIs are critical. These are challenging to target directly with small molecules and have been considered to be undruggable or, at best, very hard drug targets. Thus, for the chromosomal translocation protein LMO2, we have exploited target discovery via chromosomal translocation junctions (*38*, *39*) to target validation with scFv (*40*) and an iDAb (*9*, *23*) to drug discovery using this cell-based method. The strategy could be implemented with any other PPI of interest involving similar cell-based Abd screening. Furthermore, our work reported here, and previously (*19*), demonstrates the use of antibodies to select chemical compounds as surrogates of the antibody binding site (once considered a holy grail of antibody biology). This has recently also been shown in the case of an anti-HIV antibody (*41*). This concept can be applied to any antibody whether to extracellular, cell surface, or intracellular targets.

In conclusion, our methodology provides at least three application features. Antibody combining sites can be used for selecting chemical compounds, the method can be applied in cells to previously considered undruggable targets such as transcription factors and can be applied to drug discovery to chromosomal translocation proteins previously considered to be difficult-to-target proteins.

## MATERIALS AND METHODS

### Cell culture

HEK293T cells were grown in Dulbecco’s modified Eagle’s medium (DMEM) (Life Technologies) and supplemented with 10% fetal bovine serum (FBS) (Sigma-Aldrich) and 1% penicillin/streptomycin (PS) (Life Technologies). Cells were grown at 37°C with 5% CO_2_.

### Molecular cloning

iDAb LMO2 mutations were generated by polymerase chain reaction (PCR) site-directed mutagenesis using pEF-GFP^2^–iDAb LMO2_dm_ as template (*25*) (i.e., iDAb LMO2 S55A/T107A). The following mutations were introduced: iDAb LMO2 S28G/H31G/S55A/T107A, iDAb LMO2 S55A/E102A/T107A, iDAb LMO2 S55A/E102A/S103A/T107A, iDAb LMO2 S28G/H31G/S55A/E102A/T107A, iDAb LMO2 S28G/H31G/S55A/S103A/T107A, and iDAb LMO2 S28G/H31G/S55A/E102A/S103A/T107A (fig. S2).

LMO2 cDNA was cloned into the pEF-RLuc8-MCS and pEF-MCS-RLuc8 plasmids, and MAX bHLH (amino acids 37 to 102) was inserted into pEF-MCS-RLuc8 plasmid. iDAb LMO2, mutants iDAb LMO2, and full-length LDB1 were cloned into pEF-GFP^2^-MCS plasmid, and full-length TAL1 and cMYC bHLH (amino acids 354 to 439) were cloned into pEF-MCS-GFP^2^ plasmid. pEF-BOS-E47 plasmid was described elsewhere (*31*).

### BRET2 titration curves and competition assays

For all BRET experiments (titration curves and competition assays), 650,000 HEK293T were seeded in each well of six-well plates. After 24 hours at 37°C, cells were transfected with a total of 1.6 μg of DNA mix, containing the donor + acceptor ± competitor plasmids, using Lipofectamine 2000 transfection reagent (Thermo Fisher Scientific). For the BRET donor saturation assays, cells were transfected with 0.05 μg of donor (LMO2) and with an increased amount of acceptor plasmid (0.025, 0.05, 0.1, 0.25, 0.5, 0.75, and/or 1 μg of DNA) equalized to a total amount of 1.6 μg of DNA with an empty vector pEF-cyto-myc. In dose-response competition experiments, competitors were transfected with the following amount of DNA: 0.1, 0.5, and 1 μg. In single-dose competition experiments, competitors were transfected with 0.1 μg of DNA. Cells were detached 24 hours later, washed with phosphate-buffered saline (PBS), and seeded in a white 96-well plate (clear bottom, PerkinElmer, catalog no. 6005181) in Opti-MEM without phenol red medium complemented with 4% FBS, and cells were incubated for an additional 20 to 24 hours at 37°C before the BRET assay reading. A detailed BRET protocol is provided elsewhere (*42*).

### Cell treatment

Compounds were prepared in 100% DMSO at 10 mM. For BRET competition assays, cells were treated with the indicated compounds at concentration of 1 (or 5), 10, and 20 μM for 22 hours. For BRET-based dose-response experiments, cells were treated with compounds at concentration of 0.01, 0.1, 1, 4, 10, 25, and 50 μM for 22 hours. The compounds were diluted in the BRET medium [Opti-MEM without phenol red (Life Technologies)] supplemented with 4% FBS and with a final concentration of 0.2% DMSO.

### BRET2 measurements

BRET2 signal was determined immediately after injection of coelenterazine 400a substrate (10 μM final concentration) to cells (Cayman Chemicals) using a CLARIOstar instrument (BMG Labtech) with a luminescence module. Total GFP^2^ fluorescence was detected with excitation and emission peaks set at 405 and 515 nm, respectively. Total RLuc8 luminescence was measured with the luminescence 400- to 700-nm wavelength filter.

The BRET signal or BRET ratio corresponds to the light emitted by the GFP^2^ acceptor constructs (515 nm ± 30) upon addition of coelenterazine 400a divided by the light emitted by the RLuc8 donor constructs (410 nm ± 80). The background signal is subtracted from that BRET ratio using the donor-only negative control where only the RLuc8 fusion plasmid is transfected into the cells. The normalized BRET ratio is the BRET ratio normalized to a negative control (iDAb control or DMSO control) during a competition assay. Total GFP^2^ and RLuc8 signals were used as a proxy to ensure that similar protein expression between comparable probes were used in BRET experiments.

### Western blot analysis

Cells were washed once with PBS and lysed in SDS-tris buffer [1% SDS and 10 mM tris-HCl (pH 7.4)] supplemented with protease inhibitors (Sigma-Aldrich) and phosphatase inhibitors (Thermo Fisher Scientific). Cell lysates were sonicated with a Branson Sonifier, and the protein concentrations were determined by using the Pierce BCA (bicinchoninic acid) Protein Assay Kit (Thermo Fisher Scientific). Equal amounts of protein (20 μg) were resolved on 12.5% SDS–polyacrylamide gel electrophoresis and subsequently transferred onto a polyvinylidene fluoride membrane (GE). The membrane was blocked with 10% nonfat milk (Sigma-Aldrich) in tris-buffered saline (TBS)–0.1% Tween 20 and incubated overnight with primary antibody at 4°C. After washing, the membrane was incubated with horseradish peroxidase (HRP)–conjugated secondary antibody for 1 hour at room temperature (RT; 22°C). The membrane was washed with TBS–0.1% Tween and developed using Clarity Western ECL Substrate (Bio-Rad) and CL-XPosure films (Thermo Fisher Scientific) or the ChemiDoc XRS+ imaging system (Bio-Rad). Primary antibodies include anti-LMO2 (1:1000; R&D Systems, catalog no. AF2726), anti-GFP (1:500; Santa Cruz Biotechnology, catalog no. sc-9996), anti-biotin [1:1000; Cell Signaling Technology (CST), catalog no. 5597S], anti–β-actin (1:5000; Sigma-Aldrich, catalog no. A1978), and anti–α-tubulin (1:2000; Abcam, catalog no. ab4074). Secondary antibodies include anti-CMYC HRP-linked (Novus Biologicals, catalog no. NB600-341), anti–His-HRP (Sigma-Aldrich, A7058), anti-mouse immunoglobulin G (IgG) HRP-linked (CST), anti-rabbit IgG HRP-linked (CST), and anti-goat IgG HRP-linked (Santa Cruz Biotechnology).

### High-throughput chemical screening with LMO2-iDAb LMO2 mutant BRET biosensor

The screen was carried out in 384-well plate format. An in-house library of 10,720 compounds (comprising 6991 compounds from BioFocus and 3729 from ChemBridge) were in 96-well plate format. The library was compressed into 384-well plate format for the HTS purpose. The volume and quantities indicated are for 40 assay 384-well plates. The screen was carried out in duplicate at 10 μM. Two sessions of HTS, containing 5360 compounds each, were screened in 68 assay plates (34 assay plates in duplicate).

Before starting, HEK293T cells were seeded into 2xT175 flask. Three days later, the 2xT175 were split into 6xT175.

1) Day 1: Cell seeding. Cells were harvested from 6xT175 flasks at ~70% confluency. The cells were resuspended in 110 ml of complete DMEM, 120 × 10^6^ cells were inoculated into each of two Corning HYPERFlask M cell culture vessels (Corning, catalog no. 10030), and 560 ml of medium was added to fill one HYPERFlask.

2) Day 2: Cell transfection with pEF-LMO2-RLuc8 and pEF-GFP^2^-iDAb LMO2_dm3_. For each HYPERFlask, 10 ml of Opti-MEM was added together with 19 μg of pEF-LMO2-RLuc8, 37 μg of pEF-GFP^2^-iDAb LMO2_dm3_, and 244 μg of pEF-empty-cyto-myc plasmids. Seven hundred fifty microliters of Lipofectamine 2000 was added in 10 ml of Opti-MEM and mixed gently. The 10 ml of DNA dilution was added and incubated for 20 min. The DNA/Lipofectamine 2000 mix was added in 500 ml of complete DMEM, and the medium of the HYPERFlask had been removed. Last, the medium + transfection mix was carefully poured into the HYPERFlask without creating any bubbles, and the flask was filled with medium.

3) Day 3: Cell seeding in 384-well plates. The cells were harvested with 100 ml of trypsin that were added per HYPERFlask and incubated for 2 min at 37°C, and the trypsinized cells were transferred to a beaker containing 100 ml of complete DMEM. Each flask was washed once with 100 ml of complete DMEM and mixed gently but thoroughly to ensure single-cell suspensions (final volume for one flask: 300 ml). A total of 90 × 10^6^ cells were added per 250-ml Corning centrifuge tube (4 × 250 ml centrifuge tubes were used, which was 360 × 10^6^ transfected cells in total), and the cells were centrifuged at 220*g* for 5 min at RT. Each cell pellet (4 in total) was gently resuspended in 200 ml of Opti-MEM without red phenol + 4% FBS + 1% PS (hereafter called BRET medium) to a final concentration of 0.45 × 10^6^ of cells/ml. The cells were seeded in white 384-well plates (clear bottom, PerkinElmer, catalog no. 6007480) with a PerkinElmer Janus liquid handling workstation housed in a category 2 enclosure (45 μl per well; 20,000 cells). A blank plate was first used to remove any air bubble in the liquid handling workstation.

4) Day 3: Library dilution. Stock solutions (100 μM) were prepared for each compound in the library (the initial concentration of the library was 10 mM). One hundred fifty nanoliters of each compound (10 mM) was added using an Echo Acoustic Dispenser (Labcyte) into 15 μl of BRET medium, giving a final concentration of 100 μM.

5) Day 3: Compounds addition: A 1% DMSO was prepared in BRET medium. Five microliters of 1% DMSO solution was dispensed in the columns 1 and 2 and 23 and 24 as negative controls. Compounds were added to cells in 5 μl (100 μM) in each well using the PerkinElmer Janus liquid handling workstation (final concentration of 10 μM, 0.1% DMSO), and the plates were incubated for 20 hours.

6) Day 4: Plate reading. A PHERAstar FSX plate reader (BMG Labtech) was used to read the plates equipped with a BRET2 optic module. The GFP^2^ signal of each plate was first measured to assess the relative cell number in each well. After the GFP^2^ reading, the bottom of each plate was covered with a white tape. Eighty milliliters of 100 μM BRET substrate (i.e., coelenterazine 400a, Cayman Chemicals) was prepared by dissolving 3 mg of coelenterazine 400a in a 32 ml of 100% ethanol, and the volume was brought to 80 ml by adding 48 ml of BRET medium. BRET reading was carried out by adding 5.5 μl of coelenterazine 400a (final concentration of 10 μM) using injectors and reading the BRET signal of each well. The reading time for one 384-well plate was about 8 min. Therfore, it will take ~4.5 hours to read 34 plates.

### Purification of scFv-LMO2 for PAL analysis

For coexpression of recombinant LMO2 and anti–LMO2 scFv, the scFv was cloned into an existing bicistronic expression vector [pRK-His-TEV-VH576-LMO2; (*23*)]. DNA encoding the scFv was amplified by PCR and cloned into the pRK vector to replace the VH576 using Nco I and Eco RI restriction sites. Plasmid DNA was transformed into *E. coli* C41 (DE3) cells for protein coexpression. A single colony was used to inoculate 50 ml of LB media containing ampicillin (100 μg ml^−1^) that was grown overnight at 37°C with shaking at 225 rpm. The overnight seed culture was diluted 1:100 in 8 × 1 liter of LB containing ampicillin (100 μg ml^−1^). The cultures were grown at 37°C with shaking at 225 rpm until an OD_600_ (optical density at 600 nm) of 0.6 was reached. ZnSO_4_ was added before induction to a final concentration of 0.1 mM. Protein expression was induced by the addition of 0.5 mM isopropyl 1-thio-β-d-galactopyranosid, and the cells were incubated overnight at 16°C with shaking at 225 rpm. Cells were harvested by centrifugation at 6000 rpm for 20 min at 4°C. Cell pellets were resuspended in lysis buffer [20 mM tris (pH 8.0), 250 mM NaCl, 20 mM imidazole, 0.1 mM ZnSO_4_, 5 mM 2-mercaptoethanol, and 5% glycerol] containing EDTA-free protease inhibitor cocktail tablets (Roche, Germany) before lysis at 25 kpsi at 4°C using a cell disruptor system (Constant Systems Ltd., UK). The cell lysate was incubated with deoxyribonuclease I and 2 mM MgCl_2_ for 20 min at RT before being clarified by centrifugation at 22,000 rpm for 1 hour at 4°C. LMO2 and anti–LMO2 scFv were copurified using a 5-ml HisTrap HP column (GE Healthcare, UK) using a 50 ml of imidazole gradient from 20 to 300 mM. The protein was concentrated to 1.5 ml and purified further by gel filtration using a HiLoad 16/600 Superdex 75 column (GE Healthcare, UK) in 20 mM tris (pH 8.0), 250 mM NaCl, and 1 mM dithiothreitol. The copurification of LMO2 and anti–LMO2 scFv was verified by standard Western blotting using anti-LMO2 (R&D Systems, AF2726) and anti–His-HRP (Sigma-Aldrich, A7058).

### PAL pulldown

Abd-L26 (20 μM) with or without Abd-L9 (100 μM) (competitor) is added in a final volume of 400 μl of PBS with 40 μg of purified protein of interest (scFv-LMO2). The same samples are prepared for the no UV controls. The samples are incubated for 25 min at RT. The samples to be cross-linked are put into ice and under the UV lamp for cross-linking for 1 hour. The no UV controls are kept on ice. During the 1 hour of cross-linking, the agarose monomeric avidin beads (catalog no. 20228, Thermo Fisher Scientific) are washed twice with PBS. After the cross-link, 20 μl of washed beads is added in all the samples (cross-linked and non–cross-linked) and incubated for 2 hours at 4°C on a roller. Two hours later, the beads were washed three times with 400 μl of PBS. The samples are lastly denatured with 50 μl of 2× loading buffer with BME (betâ-mercaptoethanol) added directly on the beads (and boiled at 100°C for 5 min) and loaded for a Western blot analysis.

### CACO-2 assay

Caco-2 apparent permeability (Papp) was determined in the Caco-2 human colon carcinoma cell line as described (*43*). Cells were maintained (DMEM with 10% FBS, penicillin, and streptomycin) in a humidified atmosphere with 5% CO_2_/95% air for 10 days. Cells were plated out onto a cell culture assembly plate (Millipore, UK), and monolayer confluency was checked using a TEER (Transepithelial/endothelial Electrical Resistance) electrode before the assay. Media was washed off and replaced with Hanks’ balanced salt solution (HBSS) buffer (pH 7.4) containing compound (10 μM, 1% DMSO) in the appropriate apical and basal donor wells. HBSS buffer alone was placed in acceptor wells. In particular instances, a specific P-gp inhibitor, LY335979 (5 μM; named “inhibitor” in the column compound), was added to the HBSS to confirm that the cells are expressing functional efflux transporter proteins. The Caco-2 plate was incubated for 2 hours at 37°C. Samples from the apical (A) and basolateral (B) chambers were analyzed using a Waters (Milford, MA, US) TQ-S liquid chromatography–tandem mass spectrometry (LC-MS/MS) system. The cell permeability properties of Abd-L compounds were compared to low (nadolol) and high (antipyrine) permeability compounds and a compound with high export (indinavir).

Apparent permeability (*Papp*) was determined as follows

$$P_{\mathrm{app}}=\frac{\mathrm{Vr}\;(\text{ml})\times \mathrm{rate}\;\mathrm{of}\;\mathrm{diffusion}\;(\mu M\;s^{-1})}{A\;({\text{cm}}^{2})\times \mathrm{Co}\;(\mu M)}$$

where *Vr* is the volume of receptical, *A* is surface area of monolayer, and *Co* is the initial compound concentration in donor

### PAMPA assay

The PAMPA was used to determine compound permeability by passive diffusion. The assay used an artificial membrane consisting of 2% phosphatidyl choline in dodecane (Sigma-Aldrich, Dorset, UK). The donor plate was a MultiScreen-IP Plate with 0.45-μm hydrophobe Immobilon-P membrane (Millipore, UK), and the acceptor plate was a MultiScreen 96-well Transport Receiver Plate (Millipore, UK). The permeability was measured at three different pH levels (pH 5, 6.5, and pH 7.4) in buffer containing 1% bovine serum albumin (Sigma-Aldrich, Dorset, UK). A 10 mM DMSO stock solution of test compound was used to prepare the 10 μM PAMPA donor solutions and calibration curves in each of the three buffers.

Six microliters of the membrane solution was added to each well of the donor plate. Buffer donor solutions (200 μl) were added to the appropriate wells of the PAMPA donor plate. Three hundred microliters per well of blank PBS (pH 7.4) was added to the PAMPA acceptor plate.

The donor and acceptor plates were then sandwiched together, covered with a lid, and incubated at 30°C in a humid environment for 16 hours. After the incubation period, the plates were removed from the incubator and the sandwich was dismantled. Samples were then transferred into a fresh plate and centrifuged. All sample supernatants were diluted and analyzed using a Waters (Milford, MA, US) TQ-S LC-MS/MS system.

Permeability values (cm/s) were calculated using the following equation

$$P_{\text{app}}=C\times -\text{ln}(1-\frac{\left[{\text{drug}}_{\text{acceptor}}\right]}{{\text{drug}}_{\text{equilibrium}}})$$

$$\text{where}\;C=\frac{V_{D}\times V_{A}}{(V_{D}\times V_{A})\times \text{area}\times \text{time}}$$

where *V*_D_ is the volume of donor, *V*_A_ is the volume of acceptor, and area is the surface area of the membrane × porosity.

### Chemical synthesis

All solvents and reagents were used as supplied (analytical or high-performance liquid chromatography grade) without prior purification. Water was purified by an Elix UV-10 system. Thin-layer chromatography was performed on aluminum plates coated with 60 F254 silica. Plates were visualized using UV light (254 nm) or 1% aq. KMnO_4_. Flash column chromatography was performed on Kieselgel 60M silica in a glass column. Nuclear magnetic resonance spectra were recorded on Bruker Avance spectrometers (AVII400, AVIII 400, AVIIIHD 600, or AVIII 700) in the deuterated solvent stated. The field was locked by external referencing to the relevant deuteron resonance. Chemical shifts (δ) are reported in parts per million (ppm) referenced to the solvent peak. ^1^H spectra reported to two decimal places, ^13^C spectra reported to one decimal place, and coupling constants (*J*) are quoted in hertz (reported to one decimal place). The multiplicity of each signal is indicated by s (singlet), br. s (broad singlet), d (doublet), t (triplet), q (quartet), dd (doublet of doublets), td (triplet of doublets), qt (quartet of triplets), or m (multiplet). Low-resolution mass spectra were recorded on an Agilent 6120 spectrometer from solutions of MeOH. Accurate mass measurements were run on either a Bruker MicroTOF internally calibrated with polyalanine or a Micromass GCT instrument fitted with a Scientific Glass Instruments BPX5 column (15 m by 0.25 mm) using amyl acetate as a lock mass by the Mass Spectrometry Department of the Chemistry Research Laboratory, University of Oxford, UK; mass/charge ratio values are reported in daltons. The detailed chemical synthesis protocols and ^1^H and ^13^C spectra for the final Abd compounds (Abd-L5 to -L27) are shown in the Supplementary Materials.

## SUPPLEMENTARY MATERIALS

Supplementary material for this article is available at http://advances.sciencemag.org/cgi/content/full/7/15/eabg1950/DC1

## Appendix

View/request a protocol for this paper from *Bio-protocol*.
